# Evaluation of Small and Large Language Models for Calculation of the ASA Score and Charlson Comorbidity Index in Orthopedic Surgical Patients: A Retrospective Concordance Analysis

**DOI:** 10.3390/bioengineering13080936

**Published:** 2026-08-19

**Authors:** Marco Di Maio, Giorgio Stopper, Vincenzo Di Matteo, Katia Chiappetta, Guido Grappiolo, Mattia Loppini

**Affiliations:** 1 Department of Biomedical Sciences, Humanitas University, Via Rita Levi Montalcini 4, Pieve Emanuele, 20072 Milan, Italy; di-maio@hotmail.it (M.D.M.); vincenzo.dimatteo@humanitas.it (V.D.M.); 2 IRCCS Humanitas Research Hospital, Via Manzoni 56, Rozzano, 20089 Milan, Italy; katia.chiappetta@humanitas.it (K.C.); guido.grappiolo@me.com (G.G.); 3 School of Medicine, Degree Course in Medicine and Surgery, Humanitas University, Via Rita Levi Montalcini 4, Pieve Emanuele, 20072 Milan, Italy; giorgio.stopper@st.hunimed.eu

**Keywords:** ASA physical status, Charlson comorbidity index, large language models, small language models, pre-operative risk stratification, orthopedic surgery, inter-rater agreement, clinical natural language processing, on-premise deployment, health data privacy

## Abstract

Background: The ASA Physical Status (ASA-PS) classification and the Charlson Comorbidity Index (CCI) are common pre-operative scoring tools. Language models could automate structured pre-operative scoring, but direct comparisons require paired inference because all models are evaluated on the same patients. Methods: In this retrospective single-center concordance analysis, 101 consecutive adult orthopedic patients were independently rated by two clinicians; the rounded mean for ASA-PS and arithmetic mean for CCI formed a clinician-derived composite reference. The cohort contained no ASA-PS IV-V patients. Six model configurations received identical prompts. Agreement was assessed using quadratic weighted kappa, ICC(2,1), exact and adjacent agreement, MAD, RMSE, and Bland–Altman limits. Post hoc between-model comparisons used 10,000 patient-level paired bootstrap replicates with Benjamini–Hochberg correction. Results: Inter-clinician weighted kappa was 0.713 for ASA-PS and 0.914 for CCI. GPT-5.2 reached kappa 0.884 for ASA-PS and 0.970 for CCI. In paired analyses, GPT-5.2 had significantly higher quadratic weighted kappa than every other tested model for both outcomes and significantly higher ICC for CCI after multiplicity correction. Phi4 and deepseek-r1-70B were not significantly different from inter-clinician agreement for CCI kappa or ICC; equivalence was not tested. Conclusions: Among the six evaluated model configurations, GPT-5.2 achieved significantly higher agreement with the clinician-derived composite reference than the other tested models for both ASA-PS and CCI in post hoc paired analyses with multiplicity correction. Locally deployable phi4 and deepseek-r1-70B showed CCI agreement estimates that were not statistically distinguishable from inter-clinician agreement, although equivalence was not tested. These findings are limited to the evaluated models and study cohort.

## 1. Introduction

Pre-operative risk stratification is the first step in surgical planning and is usually performed by the anesthetist. Other steps of the preoperative evaluation include informed consent and case-mix adjustment in benchmarking and research [1,2]. Two prominent tools are mostly used to carry out this task: the ASA Physical Status (ASA-PS) classification and the Charlson Comorbidity Index (CCI) [1,3,4]. Orthopedic surgery is among the highest-volume surgical specialties, and the combination of rising volumes and marked clinical heterogeneity makes accurate pre-operative stratification an increasingly central concern and motivates the use of validated peri-operative risk-scoring models [5,6].

The ASA-PS classification, introduced in 1941 and refined since, grades patients from class I (normal healthy) to V (moribund), with 2014 vignette examples added to mitigate subjective interpretation [7]. It is the single most commonly recorded comorbidity descriptor in arthroplasty registries [1] and a core input to the ACS NSQIP surgical risk calculator [2], stratifying in-hospital mortality from 1.8% (ASA I–II) to 16.5% (ASA IV) [8]. Yet ASA-PS shows well-documented inter-rater variability, with quadratic weighted kappa typically between 0.4 and 0.7 depending on case-mix, rater experience, and availability of the 2014 examples [9,10,11]. Inter-rater agreement, not single-rater accuracy, is therefore the realistic ceiling for any AI-assisted ASA workflow.

The CCI, developed in 1987 and statistically re-weighted by Quan et al. in 2011, aggregates weighted comorbidities into a single integer score validated in joint-arthroplasty and hip-fracture populations, where higher scores predict mortality, readmission and greater resource use [3,4,12]; it remains among the most widely used orthopedic comorbidity instruments, although comparative studies show it can be outperformed by the Elixhauser measure for outcome discrimination [13,14,15]. Manual calculation is time-consuming (5–15 min per patient) and sensitive to documentation quality, with disagreement concentrated on definition-sensitive conditions; inter-rater variability is much lower for the aggregate score than for individual items [4,16,17]. ASA-PS and CCI are only moderately correlated and encode complementary constructs [18], which makes them a natural joint target for language-model evaluation along two distinct axes: coarse ordinal judgment (ASA-PS) and structured extraction over a closed list (CCI).

Both scores can be derived from narrative clinical text, a property that makes them well suited to language-model processing [19,20]. Modern transformer models [20] vary in parameter count, architecture, training pipeline, reasoning configuration, and deployment mode. Labels such as small, medium, and large are used descriptively in this study, but parameter count is confounded with these other characteristics and cannot be interpreted as an isolated exposure. Locally deployable open-weight models may permit clinical text to remain within institutional infrastructure, whereas externally hosted closed-weight models require transmission to cloud APIs, with attendant governance considerations under the GDPR and the EU AI Act [19,21]. Prior work has evaluated GPT-3.5/GPT-4-class models and dedicated NLP pipelines on ASA-PS [22,23,24,25], reporting exact agreement of roughly 50–80% and weighted kappa approaching physician–physician agreement [22,23,24], and has documented strong language-model performance on structured clinical extraction [19,21,26]. However, a same-cohort comparison of multiple model configurations using identical prompts has not been reported.

## 2. Materials and Methods

This was a retrospective, single-center, observational concordance analysis comparing language-model-based assignment of ASA-PS and CCI against a clinician-derived composite reference in adult patients undergoing orthopedic surgery at the Departments of Orthopaedic Surgery and Anaesthesiology of IRCCS Humanitas Research Hospital (Milan, Italy), using clinical records of patients admitted in 2021. The prespecified primary objective was to quantify agreement for six evaluated model configurations under identical prompts and inputs. The protocol is part of the Humanitas registry of orthopedic surgical procedures (Institutional Review Board approval no. 618/17).

Eligible patients were adults (≥18 years) admitted for orthopedic surgery with pre-operative documentation sufficient to reconstruct both scores from narrative text alone (at minimum, a pre-operative anesthesia consultation note documenting past, present and remote history, active comorbidities and current medications). Patients were excluded for incomplete documentation, procedures performed under local anesthesia without an anesthesia consultation, or records containing post-operative documentation that could leak outcome information. The analytical cohort comprised 101 consecutive patients; the sampling frame was the operating-room register, so patients whose surgery was canceled or postponed are not represented. No ASA-PS IV or V patients were included. Sample size was determined a priori on feasibility grounds. As a precision check, assuming two raters, unweighted Cohen’s kappa, five response categories, an anticipated kappa of 0.70, and approximately balanced category frequencies, 101 cases were expected to yield an approximate 95% confidence interval half-width of about 0.10.

Two early-career orthopedic surgeons, both of whom had recently completed specialist training in Orthopedics and Traumatology, independently reviewed all de-identified cases and assigned both the ASA-PS classification and the full 17-item CCI using a predefined data-collection form. At the time of the assessments, the two raters had 1 year and 2 years of post-specialty clinical experience, respectively. They were blinded to each other’s assignments and to all model outputs. Their paired ratings were used to quantify observed inter-clinician agreement and to contextualize model agreement; they were not treated as an independent external comparator for a superiority claim.

The prespecified primary comparator was a two-rater composite reference, calculated as the rounded mean of the two ASA-PS ratings using the half-up rule and the arithmetic mean of the two CCI ratings. Selecting either clinician as the primary comparator would have made the estimated model agreement contingent on an arbitrary rater choice. The two-rater composite reference was not considered an objective ground truth or an adjudicated expert consensus.

Six model configurations were selected pragmatically to include locally deployable open-weight and externally hosted closed-weight options available to the investigators when the evaluation set was established, across a range of parameter counts and inference approaches. The evaluated configurations were phi4 (Microsoft Research, 14 B parameters) [27], gemma3 (Google DeepMind, 27 B) [28,29], deepseek-r1-70B (distilled version from the actual model), GPT-5.2 with the high-reasoning configuration, o1-mini, and GPT-4o-mini. Selection was not systematic or exhaustive and was not designed to estimate a causal effect of parameter count or model class; other model families and within-family size variants were outside the fixed evaluation set. In this study, locally deployable means that inference can be performed within institutional infrastructure without transmitting clinical text to an external API with reasonable speed, except for DeepSeek 70B, which required offloading to RAM and consequently had slower token/sec. The workstation had the following specifications: CPU Intel 14th gen i7, RAM DDR5 128 GB, GPU Nvidia 3090ti, 2 TB SSD. Local models were run using Ollama Desktop version 0.19.0, with automated memory offloading when required. Output-generation throughput differed substantially among the locally deployed models. Phi4 achieved the highest generation speed at 72.91 tokens/s, followed by gemma3 at 6.81 tokens/s and deepseek-r1-70B at 1.61 tokens/s. The lower throughput observed for deepseek-r1-70B coincided with the need to offload part of the model to system RAM. These measurements are specific to the tested hardware and deployment configuration and represent output-token generation throughput rather than end-to-end response latency; therefore, they should not be interpreted as establishing real-time clinical feasibility. The context window of every model accommodated the longest clinical text. All models were queried using deterministic settings, and model identifiers, prompts, raw outputs, and timestamps were logged. For cloud-based testing, only fully anonymized text was transmitted, and data privacy settings were configured to prevent use of the data for model training.

For each patient, a structured case was assembled from five pre-operative anesthesia consultation fields (past medical history, history of present illness, pathological history, active comorbidities and current medications), transcribed verbatim into a single plaintext document. All direct identifiers (name, date of birth, medical record number, address, contacts and treating-clinician names) were removed manually during transcription, in compliance with EU Regulation 2016/679 (GDPR). Documents generated after the index surgery were excluded to avoid information leakage; no case exceeded any model’s context window.

Two prespecified system prompts (one per score) were issued identically to all six models with no model-specific tuning; the full verbatim text and JSON schemas are available from the corresponding author on request. The ASA-PS prompt (in Italian, matching the case) instructs the model to perform an internal structured reasoning step before returning a single ASA class (constrained to 1–5) with a brief explanation as a single JSON object, using the tie-breaking rule “choose the highest justified class, or in case of doubt the lowest”. The CCI prompt (in English) asks the model to decide, for each of the 17 conditions of the original Charlson index, whether the case contains evidence of that condition, distinguishing unilevel (single Boolean) from graded conditions (worst level present); the model returns a Boolean vector. The integer CCI total was not requested from the model but computed by a deterministic post-processing algorithm applying the Quan-updated 2011 weights with the standard age-adjustment (one point per decade above 50), applied identically to model outputs and to clinician ratings. JSON outputs were parsed by a strict parser.

Three prespecified stratifications were performed for both scores using the composite reference: ASA severity (low, I–II vs. elevated, III–V); CCI severity (0; moderate, 0.5–2; high, >2); and a case-complexity proxy (low/intermediate/high, combining the number of recorded comorbidities and the clinical-text length). Because chance-corrected statistics behave paradoxically in highly homogeneous strata owing to prevalence and range-restriction effects [30], every subgroup analysis reports the full panel of complementary metrics alongside kappa and ICC and notes the homogeneity of the stratum [31].

### 2.1. Outcomes

The primary outcome was the agreement between each model and the two-rater composite reference across the full cohort: the quadratic weighted Cohen’s kappa for ASA-PS [32], and both the quadratic weighted kappa and the intraclass correlation coefficient ICC(2,1) for CCI. Each primary estimate is reported with a 95% confidence interval derived by non-parametric bootstrap (2000 replications). Secondary outcomes were exact agreement (proportion of identical scores), adjacent agreement (within one class), mean absolute difference (MAD), root mean square error (RMSE), mean signed bias, and 95% Bland–Altman limits of agreement.

### 2.2. Statistical Analysis

Analyses were performed using R version 4.5.3, with readxl version 1.4.5 and openxlsx version 4.2.8.1. Agreement and paired-bootstrap functions were implemented in the accompanying R analysis script. Continuous variables are summarized as medians with ranges or interquartile ranges (IQR); categorical variables as counts and percentages. Agreement was quantified with the metrics defined above [33]; ICC could not be computed where a subgroup contained completely identical scores. The model-specific 95% confidence intervals for descriptive agreement estimates used non-parametric bootstrap resampling with 2000 replications. Between-model comparisons were conducted post hoc using patient-level paired non-parametric bootstrap resampling with 10,000 replicates and a fixed random seed (20260806). In each replicate, patients were sampled with replacement while both clinician assessments and all model outputs remained linked. The composite clinical reference and agreement statistics were recalculated within every replicate. Differences in quadratic weighted kappa were calculated for ASA-PS and CCI, with additional differences in ICC(2,1) for CCI. Ninety-fifth percentile confidence intervals were obtained for the paired differences. Two-sided bootstrap *p*-values were calculated from the centered bootstrap distributions and adjusted for multiple comparisons using the Benjamini–Hochberg procedure separately within each outcome and metric family. Because these comparisons were not prespecified, they were considered post hoc exploratory analyses. Statistical significance was not inferred from overlap between separate model confidence intervals.

### 2.3. Ethics

The study used medical records in the institutional registry of orthopedic surgical procedures of IRCCS Humanitas Research Hospital (IRB approval no. 618/17) and was conducted in compliance with the Declaration of Helsinki and EU Regulation 2016/679 (GDPR). Because the analysis used fully de-identified retrospective registry data approved for secondary use, the existing IRB approval covered these analyses and individual informed consent was waived. All inputs to the cloud LLMs consisted of de-identified text, with vendor opt-out of training-data use where available; every model output is retained in an audit trail with its corresponding input and the composite reference, and the pipeline is reproducible from the recorded model versions, prompts, seeds, and software versions, in line with EU AI Act principles for high-risk healthcare AI.

## 3. Results

The analytical cohort comprised 101 adult orthopedic surgical patients with complete clinician reference data for both scores; no patients were excluded, and there were no missing values on any primary outcome. Median age was 62 years (IQR 52–73; range 20–93); 59 patients (58.4%) were male. Median BMI was 26.6 kg/m^2^ (IQR 23.5–29.8). Loco-regional anesthesia was used in 96 of 101 procedures (95.0%). The cohort was dominated by elective lower-limb arthroplasty (75 hip, 20 knee), with four other elective procedures and two trauma cases. No ASA IV or V patients were present. After composite reference rounding, the ASA-PS distribution was 8 ASA I (7.9%), 56 ASA II (55.4%), and 37 ASA III (36.6%). Median CCI was 2 (IQR 1–4). Pre-operative clinical text had a median length of 1510 characters (IQR 1015–2256). Baseline characteristics are summarized in Table 1.

For ASA-PS, the inter-clinician quadratic weighted kappa was 0.713 (95% CI 0.559–0.828), with 79.2% exact and 100.0% adjacent agreement (MAD 0.208; signed bias −0.069). For CCI, the inter-clinician weighted kappa was 0.914 (95% CI 0.877–0.952) and ICC(2,1) was 0.915 (0.875–0.948), with 67.3% exact and 90.1% adjacent agreement, MAD 0.465, RMSE 0.917, and 95% Bland–Altman limits from −1.733 to 1.871. These values describe observed agreement between the two clinicians. Model comparisons with this benchmark are contextual because the composite model reference was constructed from the same two clinician assessments.

All six models produced parseable ASA-PS assignments for all 101 cases (Table 2). GPT-5.2 achieved the highest descriptive weighted agreement (kappa 0.884, 95% CI 0.792–0.952; 91.1% exact; MAD 0.089). The remaining models ranged from kappa 0.587 (gemma3) and 0.510 (phi4) to 0.506 (o1-mini and GPT-4o-mini) and 0.369 (deepseek-r1-70B). Adjacent agreement remained between 98.0% and 100.0% across all models, indicating that disagreements were predominantly single-class shifts. Signed bias was negative for GPT-5.2, gemma3, and deepseek-r1-70B and positive for phi4, o1-mini, and GPT-4o-mini.

All six models produced parseable CCI scores for all 101 cases (Table 3). GPT-5.2 had the highest descriptive weighted agreement (kappa 0.970; ICC 0.965), followed by phi4 (0.920; 0.919) and deepseek-r1-70B (0.918; 0.914). Gemma3 reached kappa 0.825 (ICC 0.828), and o1-mini/GPT-4o-mini reached 0.724 (0.711). GPT-5.2 had the highest exact agreement (59.4%) and the lowest error (MAD 0.322; RMSE 0.574). Despite kappa estimates above 0.91, phi4 and deepseek-r1-70B reached 36.6% and 50.5% exact agreement, respectively, indicating predominantly small numerical near-misses.

In the post hoc paired analysis, GPT-5.2 had significantly higher ASA-PS quadratic weighted kappa than each of the other five models after Benjamini–Hochberg correction. Differences ranged from 0.298 versus gemma3 (95% CI 0.183 to 0.427; adjusted *p* < 0.001) to 0.515 versus deepseek-r1-70B (95% CI 0.395 to 0.644; adjusted *p* < 0.001). All GPT-5.2 between-model comparisons are reported in Table 4.

For CCI, GPT-5.2 had significantly higher quadratic weighted kappa than every other tested model after multiplicity correction. The difference was 0.050 versus phi4 (95% CI 0.027 to 0.082; adjusted *p* = 0.004) and 0.052 versus deepseek-r1-70B (95% CI 0.017 to 0.096; adjusted *p* = 0.016). The corresponding ICC differences were also significant: 0.046 versus phi4 (95% CI 0.022 to 0.078; adjusted *p* = 0.005) and 0.051 versus deepseek-r1-70B (95% CI 0.019 to 0.090; adjusted *p* = 0.012). All GPT-5.2 between-model comparisons are reported in Table 4.

GPT-5.2 showed higher agreement with the clinician-derived composite reference than the observed inter-clinician agreement, with paired difference confidence intervals excluding zero: +0.171 for ASA-PS kappa (95% CI 0.060 to 0.305; adjusted *p* = 0.015), +0.056 for CCI kappa (95% CI 0.018 to 0.090; adjusted *p* = 0.005), and +0.050 for CCI ICC (95% CI 0.018 to 0.087; adjusted *p* = 0.011). This comparison is contextual rather than an independent test of superiority over clinicians because the composite reference was derived from the same two clinician assessments. Phi4 and deepseek-r1-70B were not significantly different from the inter-clinician benchmark for CCI kappa or ICC. Equivalence was not tested.

Sensitivity analyses using each clinician separately preserved the overall ordering. GPT-5.2 had ASA-PS kappa values of 0.840 against Clinician 1 and 0.792 against Clinician 2; for CCI, its kappa values were 0.947 and 0.940, and its ICC values were 0.948 and 0.941. Across the same clinician-specific CCI analyses, phi4 and deepseek-r1-70B kappa and ICC estimates ranged from 0.879 to 0.918.

**Table 4:** Post hoc paired inferential comparisons between GPT-5.2 and each other tested model. Differences are GPT-5.2 minus comparator; positive values indicate higher agreement for GPT-5.2. Confidence intervals are patient-level paired percentile bootstrap 95% confidence intervals based on 10,000 replicates. Adjusted *p*-values use the Benjamini–Hochberg procedure separately within each outcome and metric family.

Outcome /Metric	Comparator	Difference	95% CI	Adjusted *p*
ASA-PS kappa	gemma3	0.298	0.183 to 0.427	<0.001
ASA-PS kappa	phi4	0.375	0.253 to 0.497	<0.001
ASA-PS kappa	o1-mini	0.378	0.278 to 0.476	<0.001
ASA-PS kappa	GPT-4o-mini	0.378	0.278 to 0.476	<0.001
ASA-PS kappa	deepseek-r1-70B	0.515	0.395 to 0.644	<0.001
CCI kappa	phi4	0.050	0.027 to 0.082	0.004
CCI kappa	deepseek-r1-70B	0.052	0.017 to 0.096	0.016
CCI kappa	gemma3	0.145	0.082 to 0.225	<0.001
CCI kappa	o1-mini	0.246	0.155 to 0.327	<0.001
CCI kappa	GPT-4o-mini	0.246	0.155 to 0.327	<0.001
CCI ICC(2,1)	phi4	0.046	0.022 to 0.078	0.005
CCI ICC(2,1)	deepseek-r1-70B	0.051	0.019 to 0.090	0.012
CCI ICC(2,1)	gemma3	0.137	0.075 to 0.217	0.001
CCI ICC(2,1)	o1-mini	0.254	0.174 to 0.328	<0.001
CCI ICC(2,1)	GPT-4o-mini	0.254	0.174 to 0.328	<0.001

Stratified by ASA severity, the low-severity subgroup (ASA I–II, n = 54) showed perfect inter-clinician agreement (kappa = 1.000), and GPT-5.2 also had kappa = 1.000; the other models showed slight-to-fair agreement. In the elevated subgroup (effectively ASA III only, n = 47), the inter-clinician kappa was paradoxically negative (−0.248) despite 55.3% exact and 100.0% adjacent agreement, and all six model kappa estimates collapsed to 0.000, illustrating the kappa paradox in a degenerate stratum (Table 5). For CCI, GPT-5.2 maintained weighted kappa above 0.83 in every subgroup, whereas o1-mini and GPT-4o-mini reached kappa 0.008 (MAD 2.17; RMSE 2.54) in the high-complexity stratum (Table 6). Phi4 and gemma3 had high-complexity kappa values of 0.665 and 0.271, respectively.

## 4. Discussion

In this retrospective single-center concordance study of 101 adult orthopedic surgical patients, three findings are most relevant. First, post hoc patient-level paired analyses showed that GPT-5.2 had significantly higher agreement with the clinician-derived composite reference than every other tested configuration for ASA-PS kappa, CCI kappa, and CCI ICC after Benjamini–Hochberg correction. These analyses were not prespecified and are exploratory. Second, the smallest CCI differences were observed against phi4 and deepseek-r1-70B, but they remained statistically significant. Their CCI kappa and ICC estimates were not significantly different from observed inter-clinician agreement; this nonsignificance does not establish equivalence. Third, adjacent agreement remained high across configurations, indicating that many disagreements were small rather than gross.

For ASA-PS, published clinician–clinician-weighted kappa generally falls between 0.4 and 0.7 [7,9,10]; the observed inter-clinician kappa of 0.713 was at the upper end of that range. GPT-5.2 had the highest descriptive kappa (0.884) and exceeded each other tested model by 0.298 to 0.515 in the paired analysis. Its agreement with the clinician-derived composite reference was also higher than observed inter-clinician agreement by 0.171, with a confidence interval excluding zero. This latter comparison is contextual, not an independent demonstration of superiority over clinicians, because the same clinician assessments were used to construct the composite reference. The remaining configurations produced kappa values of 0.369 to 0.587, although adjacent agreement remained 98–100%, indicating predominantly one-class shifts.

For CCI, the paired results refine the descriptive ranking. GPT-5.2 had significantly higher kappa and ICC than all other tested configurations. The smallest differences were against phi4 and deepseek-r1-70B: 0.050 and 0.052 for kappa and 0.046 and 0.051 for ICC, respectively. Phi4 and deepseek-r1-70B were not significantly different from the inter-clinician benchmark for CCI kappa or ICC, but equivalence was not tested. Clinician-specific sensitivity analyses preserved the same pattern, with GPT-5.2 remaining the highest-agreement configuration against each clinician separately. These findings support a task-specific interpretation of model performance without implying that the locally deployable models and clinicians are interchangeable.

The deployment interpretation should remain limited to data location and observed feasibility under the tested conditions. Phi4, gemma3, and deepseek-r1-70B were run locally, whereas the OpenAI configurations required external API transmission of anonymized text. Phi4 and gemma3 operated at functional generation speeds, while deepseek-r1-70B required memory offloading and was consequently slower. Accordingly, “locally deployable” indicates that inference can occur within institutional infrastructure without transmitting clinical text externally. These findings should not be interpreted as a comprehensive comparison of hardware requirements, inference speed, consumer-grade feasibility, or operational cost.

Two methodological observations dominate the subgroup analyses. First, prevalence and range-restriction effects on kappa [30] are vividly illustrated in the ASA III–V stratum, which contained only ASA III patients: the inter-clinician kappa was negative (−0.248) despite 55.3% exact and 100.0% adjacent agreement, and every model kappa collapsed to 0.000. These observations illustrate the limitations of kappa in highly homogeneous, range-restricted strata and reinforce the need to interpret kappa alongside exact and adjacent agreement. Second, GPT-5.2 was the only model that retained robust performance across all subgroups, whereas o1-mini and GPT-4o-mini collapsed in the high-complexity CCI stratum (kappa 0.008; MAD 2.17), consistent with the observation that the gap between frontier models and the rest of the field widens on tasks requiring integration across long, heterogeneous narratives [21].

Clinically, high adjacent agreement is reassuring at the population level, but a one-class ASA-PS shift can still affect peri-operative planning and case-mix adjustment. For CCI, high kappa can coexist with lower exact agreement because small numerical differences receive partial credit under quadratic weighting. The present concordance results therefore do not establish that any model can replace a clinician, improve workflow, or improve patient outcomes. The appropriate interpretation is an assistive application with clinician oversight, subject to external validation and prospective workflow evaluation [34,35].

This study has several strengths: a complete analytical cohort with no missing primary-outcome data; independent clinician ratings and a clearly defined composite reference; identical prompts and inputs across six evaluated configurations; a complementary panel of weighted, absolute, and error-based metrics; patient-level paired bootstrap comparisons with multiplicity correction; and clinician-specific sensitivity analyses. The CCI pipeline also separated concept recognition from deterministic score calculation.

The present findings apply to a relatively homogeneous, predominantly elective, single-center orthopedic cohort restricted to ASA-PS classes I–III. No ASA-PS IV–V patients were included. The results should not be extrapolated to emergency surgery, major trauma, higher-acuity populations, other specialties, languages, institutions, or documentation systems without external validation.

Limitations include the single-center retrospective design, modest sample size, and homogeneous case-mix, which produced unstable variance-dependent statistics in some subgroups. The two-rater composite reference was not an objective ground truth or adjudicated consensus, and averaging may generate a value not assigned by either clinician, particularly for CCI. Because both clinician ratings also defined the inter-clinician benchmark, model-versus-benchmark comparisons are contextual and cannot establish independent superiority over clinicians. The between-model analyses were post hoc and should be considered exploratory.

Model selection was pragmatic and non-exhaustive. Parameter count was confounded with developer, architecture, training pipeline, reasoning configuration, and deployment mode; therefore, the study cannot isolate a causal effect of scale or support generalization to SLM, MLM, or LLM classes. Output-generation throughput was recorded for the locally deployed models; however, standardized end-to-end latency and time-to-first-token were not measured, precluding a comprehensive comparison of operational feasibility. In addition, all source narratives came from one Italian institution with recurring local terminology and note structure, so performance may partly reflect adaptation to this linguistic and documentation environment. Future work should include multicenter and multilingual external validation, ASA-PS IV–V and emergency cases, independent adjudication, standardized hardware and latency benchmarking, within-family model comparisons, and prospective clinical workflow and safety outcomes. We note that o1-mini and GPT-4o-mini produced identical outputs across all metrics, which we attribute to architectural similarity between these mini-tier configurations; further investigation is warranted.

## 5. Conclusions

The cohort contained no ASA-PS IV-V patients, and external validation is required before extrapolation to higher-acuity populations. Among the six evaluated configurations, GPT-5.2 demonstrated significantly higher agreement with the clinician-derived composite reference than the other tested models for both ASA-PS and CCI. These comparisons were post hoc and should be considered exploratory. Because parameter count was confounded with developer, architecture, training pipeline, reasoning configuration, and deployment mode, the findings do not establish a causal effect of model scale and should not be generalized to SLM, MLM, or LLM tiers as model classes.

## Figures and Tables

**Table 1:** Baseline characteristics of the analytical cohort (n = 101). ASA-PS composite reference = rounded mean of the two raters (half-up); CCI composite reference = arithmetic mean of the two raters. IQR = interquartile range.

Characteristic	Value
Age median (years); [IQR]; (range)	62; [52–73]; (20–93)
Male sex, n (%)	59 (58.4%)
Female sex, n (%)	42 (41.6%)
Body mass index median (kg/m^2^); [IQR]	26.6 (23.5–29.8)
Anesthesia: loco-regional, n (%)	96 (95.0%)
Anesthesia: general/spinal/local-sedation, n	3/1/1
Procedure: hip arthroplasty, n (%)	75 (74.3%)
Procedure: knee arthroplasty, n (%)	20 (19.8%)
Procedure: other elective/trauma, n (%)	4 (4.0%)/2 (2.0%)
ASA-PS composite reference—I/II/III, n	8/56/37
ASA-PS IV–V, n	0
CCI composite reference—median (IQR)	2 (1–4)
Clinical text length, characters—median (IQR)	1510 (1015–2256)

**Table 2:** Full-cohort agreement with the two-rater composite reference, ASA Physical Status (n = 101). Quadratic weighted kappa; exact agreement is against the rounded composite reference; adjacent (Adj.) agreement is within one ASA class. Models are listed in descending order of kappa.

Comparator	Tier	Weighted Kappa (95% CI)	Exact (%)	Adj. (%)	MAD	Bias
Inter-clinician (R1 vs. R2)	clinician	0.713 (0.559–0.828)	79.2	100.0	0.208	−0.069
GPT-5.2	LLM	0.884 (0.792–0.952)	91.1	100.0	0.089	−0.089
gemma3	MLM	0.587 (0.432–0.714)	70.3	99.0	0.307	−0.089
phi4	SLM	0.510 (0.370–0.622)	67.3	100.0	0.327	+0.149
o1-mini	LLM	0.506 (0.396–0.591)	51.5	100.0	0.485	+0.406
GPT-4o-mini	LLM	0.506 (0.396–0.591)	51.5	100.0	0.485	+0.406
deepseek-r1-70B	LLM	0.369 (0.229–0.496)	51.5	98.0	0.505	−0.465

**Table 3:** Full-cohort agreement with the two-rater composite reference, Charlson Comorbidity Index (n = 101). Quadratic weighted kappa; ICC(2,1) = two-way random-effects, single-rater, absolute-agreement intraclass correlation. The per-model CCI integer is the deterministic post-processing of the model’s Boolean condition vector under Quan 2011 weights with age adjustment. Models are listed in descending order of kappa.

Comparator	Tier	Weighted Kappa (95% CI)	ICC(2,1)	Exact (%)	Adj. (%)	MAD	RMSE
Inter-clinician (R1 vs. R2)	clinician	0.914 (0.877–0.952)	0.915	67.3	90.1	0.465	0.917
GPT-5.2	LLM	0.970 (0.953–0.981)	0.965	59.4	96.0	0.322	0.574
phi4	SLM	0.920 (0.885–0.947)	0.919	36.6	90.1	0.639	0.913
deepseek-r1-70B	LLM	0.918 (0.875–0.950)	0.914	50.5	89.1	0.510	0.869
gemma3	MLM	0.825 (0.744–0.888)	0.828	46.5	81.2	0.718	1.207
o1-mini	LLM	0.724 (0.641–0.811)	0.711	42.6	78.2	0.866	1.443
GPT-4o-mini	LLM	0.724 (0.641–0.811)	0.711	42.6	78.2	0.866	1.443

**Table 5:** ASA-PS agreement by ASA severity stratum. ASA I–II n = 54; ASA III–V n = 47 (all ASA III). Quadratic-weighted kappa; exact = exact agreement against the rounded composite reference; adj. = adjacent agreement (within one ASA class). The negative inter-clinician kappa in the ASA III–V stratum reflects range restriction (kappa paradox), not poor agreement.

Comparator	Tier	ASA I–II Kappa	ASA I–II Exact (%)	ASA III–V Kappa	ASA III–V Exact (%)	ASA III–V Adj. (%)
Inter-clinician	clinician	1.000	100.0	−0.248	55.3	100.0
GPT-5.2	LLM	1.000	100.0	0.000	80.9	100.0
phi4	SLM	0.165	55.6	0.000	80.9	100.0
o1-mini	LLM	0.222	38.9	0.000	66.0	100.0
GPT-4o-mini	LLM	0.222	38.9	0.000	66.0	100.0
gemma3	MLM	0.372	75.9	0.000	63.8	97.9
deepseek-r1-70B	LLM	0.407	79.6	0.000	19.1	95.7

**Table 6:** CCI quadratic weighted kappa by subgroup, all models. Subgroup sizes: ASA I–II n = 54; ASA III–V n = 47; CCI 0 n = 17; CCI 0.5–2 n = 38; CCI > 2 n = 46; complexity low n = 29; complexity high n = 29. Values of 1.000 or 0.000 in homogeneous strata must be read alongside the absolute and error-based metrics.

Comparator	Tier	ASA I–II	ASA III–V	CCI 0	CCI 0.5–2	CCI > 2	Compl. Low	Compl. High
Inter-clinician	clinician	0.940	0.866	1.000	0.365	0.756	0.862	0.646
GPT-5.2	LLM	0.977	0.962	1.000	0.719	0.912	0.964	0.835
phi4	SLM	0.886	0.916	0.000	0.431	0.807	0.663	0.665
deepseek-r1-70B	LLM	0.963	0.857	0.000	0.652	0.685	0.893	0.396
gemma3	MLM	0.885	0.753	1.000	0.648	0.509	0.964	0.271
o1-mini	LLM	0.885	0.557	1.000	0.569	0.260	0.964	0.008
GPT-4o-mini	LLM	0.885	0.557	1.000	0.569	0.260	0.964	0.008

## Data Availability

Individual-level clinical narratives are not publicly available because of patient confidentiality and institutional data-protection restrictions. The model prompts, output schemas, R analysis code, and de-identified derived results are available from the corresponding author upon reasonable request, subject to institutional and ethical approval. The completed GRRAS checklist is provided in the Supplementary Materials.

## Supplementary Materials

The following supporting information can be downloaded at: https://www.mdpi.com/article/10.3390/bioengineering13080936/s1.
